# Sacral Rheumatoid Nodule: An Unusual Indication for Coccygectomy

**DOI:** 10.1155/2020/2757625

**Published:** 2020-05-08

**Authors:** Zachary R. Porter, Juan C. Mejia-Munne, Bryan M. Krueger, Jennifer A. Kosty, Laura B. Ngwenya

**Affiliations:** ^1^College of Medicine, University of Cincinnati, Cincinnati, Ohio, USA; ^2^Department of Neurosurgery, University of Cincinnati, USA; ^3^Department of Neurology and Rehabilitation Medicine, University of Cincinnati, USA

## Abstract

Here, we present a case report of a woman who presented with a large sacral rheumatoid nodule. This patient failed conservative treatment and presented in search of a surgical solution. We successfully removed her rheumatoid nodule using a surgical approach typically reserved for traumatic coccydynia. We show how coccygectomy, although a rare surgical procedure, was effective in treatment of a large rheumatoid nodule.

## 1. Introduction

Rheumatoid arthritis is a chronic inflammatory disease that is predominately characterized by a variety of extra-articular manifestations. The development of disease-modifying antirheumatic drugs (DMARDs) has altered the treatment paradigm of rheumatoid arthritis; however, extra-articular rheumatoid nodules (RNs) may become refractory to DMARD treatment. In such cases, RNs may progress to severe joint destruction and prevent individuals from attaining an active lifestyle. Synovectomy and nodule resection have been previously described for treatment refractory RNs in most joints. However, due to the rarity of sacral RNs, no previous cases have been reported describing surgical options for severe refractory cases. Here, we present a case of a woman with a large sacral rheumatoid nodule that caused discomfort and was resistant to conservative treatment. We present our surgical approach to removal of this nodule as well as a brief description of the surgical procedure and a review of its use for paracoccygeal masses.

## 2. Case Presentation

**Figure 1:**
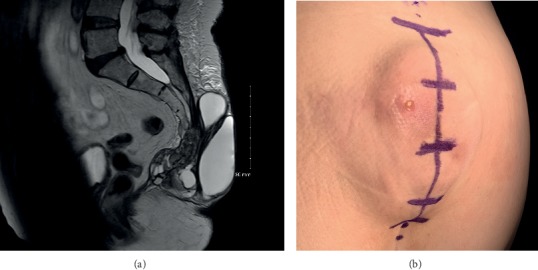
(a) T2 MRI demonstrating a large cystic lesion with a fibrous mass connected to the coccyx. (b) Intraoperative photo demonstrating large mass prior to surgical incision.

A 77-year-old woman with a history of rheumatoid arthritis with numerous nodules and a previous C1-C2 posterior fixation and fusion for atlantoaxial instability presented for evaluation with a ten-year history of a painful mass in the region between her lower back and intergluteal cleft. The mass had been intermittently drained by another physician for some time with recurrence and persistent symptoms. Computerized tomography and magnetic resonance imaging were performed which demonstrated a large, multilobulated cystic nodule in the region of the sacrococcygeal joint (Figure 1). The patient had failed the conservative treatment of intermittent drainage, and throughout the years, the need for drainage had become more frequent, with her requiring drainage of the cyst every month. With each subsequent drainage, only a small amount of fluid was able to be drained, and the patient presented to our clinic with significant pain and inquiring about a surgical solution.

**Figure 2:**
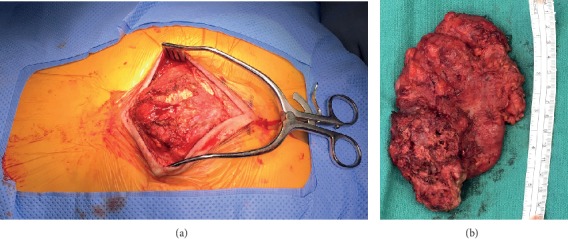
(a) Intraoperative photo showing mass after skin incision. (b) The mass and coccyx were resected en bloc as seen in this intraoperative photo.

After evaluation, the patient underwent surgery for removal of the lesion. A posterior sacrococcygeal approach was utilized for en bloc resection of the cystic nodule and coccyx (Figure 2). Plastic surgery assistance was utilized for reconstruction of the pelvic floor muscles and mobilization of the gluteus muscles and fasciocutaneous flaps. Pathology demonstrated a benign cyst with giant cells, acute and chronic inflammation, and prominent fibrosis, consistent with a rheumatoid nodule.

The patient was not able to follow up in our clinic due to transportation issues. However, in a telephone follow-up one year post surgery, she reported doing well without recurrence of the painful sacral nodule.

## 3. Discussion

Also called necrobiotic nodules, RNs are a common complication of rheumatoid arthritis and effect between 30-40% of patients [1, 2]. Risk factors for developing RN in patients with rheumatoid arthritis include male sex, younger age at disease onset, longer duration of disease, mononuclear phagocyte- (MPH-) activated immune complexes, high circulating levels of Th1 cytokine and macrophage profiles, and seropositivity for rheumatoid factor [2–6]. Most commonly found near areas of subcutaneous pressure points, the pathogenesis of RN formation is thought to be related to damage of nearby microvasculature due to repetitive injury or chronic inflammation [1, 5, 6]. Treatment of RNs usually consists of conservative management, and drainage is not recommended due to a high risk of recurrence and infection [5]. While little is known regarding treatment for coccydynia secondary to sacral RN, surgical removal of RNs in general may be necessary in cases of debilitating symptoms, nerve root compression, ulceration, or infection [5].

Coccydynia secondary to sacral RN is a rare entity with only four case reports previously described in the literature [3, 7]. Of these, 2 cases reported effective treatment following continued treatment for systemic rheumatoid arthritis, one case reported effective treatment following resection of the sacral nodule, and one case reported a patient who developed a recurrence of their sacral RN following nodule resection. This patient was treated aggressively with antibiotics and wound debridement; however, she unfortunately died after developing subsequent bronchopneumonia [3]. While no studies have been described detailing resection of sacral RNs, resection of RNs in other areas has been found to have a high rate of recurrence. Two retrospective cohorts of patients undergoing synovectomy for elbow RNs found that over half of patients developed recurrence of the RN within 5 years [8, 9]. The patient reported here also experienced multiple local RN recurrences after sacral nodule removal; however, she has retained good local control following coccygectomy. As RN is an extra-articular manifestation of an intra-articular process, it is understandable that removal of RNs often results in immediate postoperative relief with delayed local recurrence. It is feasible that the removal of the nodule and the affected joint helped the patient described here be recurrence-free as this treatment option attempted to remove the intra-articular contribution to her extra-articular disease process.

Described in 1972, the Gardner method for coccygectomy is often employed and has been associated with good results. From the posterior approach, the patient is placed in the jack-knife position. A 7.5 cm cut is made from above the sacrococcygeal joint down the lien of the buttocks. After cutting through to the coccyx, the tip of the coccyx is raised and the tip of the coccyx is cut away from the tissue around the anus. Using a wet sponge, the rest of the coccyx is separated from the tissues underneath it all the way to the sacrococcygeal joint, and the sacrococcygeal joint is transected [6]. Minor deviations to this technique have been reported. For example, Patsouras et al. utilize preoperative bowel preparation with 5 days of a minimal residue diet, an incision to the edge of the anal sphincters at 6 o'clock, and transanal digital examination to confirm the posterior rectal wall [10].

Though coccygectomy has traditionally been utilized for management of traumatic coccydynia refractory to conservative measures, this technique has been reported as useful in the treatment of paracoccygeal masses. Nonidiopathic causes of coccydynia are rare, and including the patient reported here, we found 10 case reports in the literature describing coccygectomy for nonidiopathic coccydynia. The age of 8 female and 2 male patients ranged from 20 to 77 years (mean 51.1 ± 20.6). Diagnoses included carcinoid tumor [11], paracoccygeal teratoma [12, 13], epithelial cyst [14], mature teratoma [15], sacrococcygeal chordoma [16], sacral giant cell tumor [17], rectal carcinoma metastasis [18], and benign dermoid cyst [19]. Coccygectomy was the first-line therapy in 8 patients following failure of conservative management or was performed after failure of antibiotics or chemotherapy (Table 1). All patients were described to have good outcomes without signs of recurrence at the most recent follow-up.

## Figures and Tables

**Table 1:** Case reports of patients treated for sacral nodules or other forms of nonidiopathic coccydynia.

Author/year	Age/sex	Symptom duration	Indication	Treatment	Prior treatment	Complication	Outcome
Sturrock, 1975	67/F	30 years	Nodular rheumatoid arthritis	Skin grafting	Topical antibiotics	None	Good, no recurrence
Sturrock, 1975	78/M	8 months	Nodular rheumatoid arthritis	Nodule resection	Conservative	Sacral ulcer, bronchopneumonia	Deceased, with local recurrence
Sturrock, 1975	68/F	5 years	Nodular rheumatoid arthritis	Prednisolone	Conservative	None	Good, no recurrence
Krasin, 2001	40/F	4 years	Carcinoid tumor	Coccygectomy	Chemotherapy	None	Good, no recurrence
Tulchinsky, 2005	44/F	7 years	Paracoccygeal teratoma	Coccygectomy	Antibiotics	Minor wound infection	Good, no recurrence
Gavriilidis, 2013	73/M	4 months	Sacrococcygeal chordoma	Coccygectomy	Conservative	None	Good, no recurrence
Kye, 2011	66/F		Epithelial cyst	Coccygectomy	Conservative	None	Good, no recurrence
Kye, 2011	24/F		Mature teratoma	Coccygectomy	Conservative	None	Good, no recurrence
Kye, 2011	53/F		Mature teratoma	Coccygectomy	Conservative	None	Good, no recurrence
Stewart, 2011	65/F		Rectal carcinoma metastasis	Coccygectomy	Conservative	None	Good, no recurrence
Goncalves, 2014	29/F	6 months	Sacral giant cell tumor	Coccygectomy	Conservative	None	Good, no recurrence
Kato, 2016	65/F	2 weeks	Nodular rheumatoid arthritis	Etanercept, prednisolone, methotrexate	Conservative	None	Good, no recurrence
Diaz-Aguilar, 2017	23/F		Mature sacral teratoma	Coccygectomy	Conservative	None	Good, no recurrence
Gaike, 2017	20/M	2 years	Benign dermoid cyst	Coccygectomy	Conservative	None	Good, no recurrence
Ngwenya, 2020	77/F	3 years	Nodular rheumatoid arthritis	Coccygectomy	Antibiotics, cyst drainage	None	Good, no recurrence

## Data Availability

Additional details of the case report are available from the corresponding author upon request. The data supporting the review of the literature are from previously reported studies, which have been cited.
